# What Lessons can Be Learned From the Management of the COVID-19 Pandemic?

**DOI:** 10.3389/ijph.2025.1607727

**Published:** 2025-05-30

**Authors:** Gerry A. Quinn, Ronan Connolly, Coilín ÓhAiseadha, Paul Hynds, Philipp Bagus, Ronald B. Brown, Carlos F. Cáceres, Clare Craig, Michael Connolly, Jose L. Domingo, Norman Fenton, Paul Frijters, Steven Hatfill, Raymond Heymans, Ari R. Joffe, Rosamond Jones, Gordan Lauc, Therese Lawrie, Robert W. Malone, Alan Mordue, Greta Mushet, Anton O’Connor, Jane Orient, José Antonio Peña-Ramos, Harvey A. Risch, Jessica Rose, Antonio Sánchez-Bayón, Ricardo F. Savaris, Michaéla C. Schippers, Dragos Simandan, Karol Sikora, Willie Soon, Yaffa Shir-Raz, Demetrios A. Spandidos, Beny Spira, Aristides M. Tsatsakis, Harald Walach

**Affiliations:** ^1^ Centre for Molecular Biosciences, Ulster University, Coleraine, United Kingdom; ^2^ Center for Environmental Research and Earth Sciences (CERES), Salem, MA, United States; ^3^ Independent Researcher, Dublin, Ireland; ^4^ Department of Public Health, Health Service Executive, Dublin, Ireland; ^5^ Sustainability and Health Research Hub, Technological University Dublin, Dublin, Ireland; ^6^ Irish Centre for Research in Applied Geosciences, School of Earth Sciences, College of Science, University College Dublin, Cork, Ireland; ^7^ Department of Applied Economics, Faculty of Law and Social Sciences, Rey Juan Carlos University, Móstoles, Spain; ^8^ School of Public Health Sciences, Faculty of Applied Health Sciences, University of Waterloo, Waterloo, ON, Canada; ^9^ School of Public Health and Administration, Universidad Peruana Cayetano Heredia, Miraflores, Peru; ^10^ Health Advisory and Recovery Team, London, United Kingdom; ^11^ Faculty of Medicine and Health Sciences, University of Rovira i Virgili, Reus (Tarragona), Spain; ^12^ School of Electronic Engineering and Computer Science, Queen Mary University, London, United Kingdom; ^13^ Department of Social Policy, London School of Economics, London, United Kingdom; ^14^ London Center For Policy Research, New York, NY, United States; ^15^ Independent Researcher, Koedijk, Netherlands; ^16^ John Dossetor Health Ethics Center, University of Alberta, Edmonton, AB, Canada; ^17^ Faculty of Pharmacy and Biochemistry, University of Zagreb, Zagreb, Croatia; ^18^ The Evidence-Based Medicine Consultancy Ltd., Bath, United Kingdom; ^19^ The Malone Institute, Madison, VA, United States; ^20^ Independent Researcher, Melrose, United Kingdom; ^21^ Association of American Physicians and Surgeons, Tucson, AZ, United States; ^22^ Department of Political Science and Administration, University of Granada, Granada, Spain; ^23^ School of Public Health, Yale University, New Haven, CT, United States; ^24^ Brownstone Institute, Austin, TX, United States; ^25^ Department of Applied Economics, Rey Juan Carlos University, Móstoles, Spain; ^26^ Department of Gynecology and Obstetrics, Faculty of Medicine, Federal University of Rio Grande do Sul, Porto Alegre, Brazil; ^27^ Department of Organisation and Personnel Management, Rotterdam School of Management, Erasmus University Rotterdam, Rotterdam, Netherlands; ^28^ Faculty of Social Sciences, Brock University, St. Catharines, ON, Canada; ^29^ Faculty of Medicine and Health Sciences, University of Buckingham, Buckingham, United Kingdom; ^30^ Department of Earth Sciences, Institute of Earth Physics and Space Science, Sopron, Hungary; ^31^ School of Public Health, University of Haifa, Haifa, Israel; ^32^ Laboratory of Clinical Virology, School of Medicine, University of Crete, Heraklion, Greece; ^33^ Departamento de Microbiologia, Instituto de Ciências Biomédicas, Universidade de São Paulo, São Paulo, Brazil; ^34^ Laboratory of Toxicology and Forensic Chemistry, School of Medicine, University of Crete, Heraklion, Greece; ^35^ Next Society Institute, Kazimieras Simonavičius University, Vilnius, Lithuania

**Keywords:** COVID-19 vaccination, epidemiology, mathematical modelling, COVID-19, public health

## Abstract

During the COVID-19 pandemic (2020–2023), governments around the world implemented an unprecedented array of non-pharmaceutical interventions (NPIs) to control the spread of SARS-CoV-2. From early 2021, these were accompanied by major population-wide COVID-19 vaccination programmes–often using novel mRNA/DNA technology, although some countries used traditional vaccines. Both the NPIs and the vaccine programmes were apparently justified by highly concerning model projections of how the pandemic could progress in their absence. Efforts to reduce the spread of misinformation during the pandemic meant that differing scientific opinions on each of these aspects inevitably received unequal weighting. In this perspective review, based on an international multi-disciplinary collaboration, we identify major problems with many aspects of these COVID-19 policies as they were implemented. We show how this resulted in adverse impacts for public health, society, and scientific progress. Therefore, we propose seven recommendations to reduce such adverse consequences in the future.

## Background

In December 2019, a cluster of patients with a novel acute respiratory illness was identified in Wuhan, Hubei Province, China [1]. The infectious agent causing this illness was named “severe acute respiratory syndrome coronavirus 2” (SARS-CoV-2), and the respiratory disease associated with it was dubbed “coronavirus disease 2019” (COVID-19) [2].

Early estimates of the severity of COVID-19 were highly alarming. Meanwhile, reports of the virus transmissibility were disturbingly high–indeed one case series estimated that 41% of the cases were nosocomial, arising from hospital-associated transmission [1]. It was later estimated that the incidence of post-viral fatigue and other post-infection sequelae (dubbed “long COVID-19” when associated with COVID-19) was 1.4–2 times greater than for influenza [3].

Worldwide concern rapidly increased as cases were identified in other parts of the world [4]. On 11 March 2020, the World Health Organization (WHO) declared that there was a global COVID-19 pandemic, or “Public Health Emergency of International Concern” [5].

Computer model projections based on these early estimates were alarming. They predicted that–unless major interventions were urgently implemented–most of the population would become infected within months, overwhelming hospital capacity and resulting in many deaths [6]. In response to these model projections, governments around the world quickly implemented an unprecedented array of “non-pharmaceutical interventions” (NPIs) [7–12], e.g., stay-at-home measures in a desperate effort to urgently reduce the spread of the virus. Governments modified the stringency of the NPIs throughout the pandemic–sometimes introducing new measures (e.g., the use of face masks was only introduced in mid-2020) or increasing their stringency, but other times, removing or reducing the extent of these measures.

By early 2021, several pharmaceutical companies announced that they had successfully developed COVID-19 vaccines that were safe and effective at preventing symptomatic COVID-19 illness [13–15]. Many countries began vaccination programmes, typically starting with healthcare workers and the elderly, but eventually becoming population-wide. To maximise the percentage of the population that was vaccinated, many governments introduced policies and strategies throughout 2021 and 2022 encouraging people to get vaccinated and discouraging people from remaining unvaccinated [16].

Although many countries had reached high levels of COVID-19 vaccination by mid-to-late 2021, the incidence of COVID-19 continued among both vaccinated (“breakthrough cases”) and unvaccinated [17–20]. However, many speculated that–even if the vaccines might be less effective against preventing infection than originally thought–they might potentially reduce the severity of disease and/or the chances of death [18, 21, 22]. Hence, the population-wide vaccination programmes were continued, and previously vaccinated people were encouraged to take additional “booster” doses [16, 18, 19].

Finally, on 5 May 2023, the WHO declared that while COVID-19 was “here to stay,” it was no longer a “global health emergency” [23]. Hence, over subsequent weeks and months, governments that had not already done so proceeded to lift remaining NPIs or vaccine requirements.

Since this declaration, the world is still coming to terms with the consequences of both the pandemic and the responses to the pandemic. However, in our opinion, there is already enough data and evidence to show that there were significant opportunities for improvement with the latter, i.e., how the world collectively responded to the COVID-19 pandemic.

Indeed, during the pandemic, each of us raised serious concerns about at least one aspect of the general thinking that apparently underpinned COVID-19 responses. We have also observed an increasing number of papers whose findings contradict multiple claims from earlier in the pandemic. This suggests that the science was not as definitive as was asserted at the time [24–27]. We are also alarmed at the way in which science has been overly tied to politics during the pandemic, constraining its flexibility. With the benefit of hindsight, we can see that genuine scientific inquiry into complex and multi-faceted research questions was inadvertently compromised under the initially laudable goal of “fighting scientific misinformation” [28–30].

Hence, we would hope that in future there can be more recognition from the scientific and medical communities, policymakers and the wider public that many of the policies that were followed during the pandemic might: a) have had serious flaws; b) have involved mistaken assumptions; or c) simply been wrong.

We note that often explicit concerns and/or warnings about various aspects of these policies had already been published in the scientific literature, i.e., were part of the published science available at the time. As you read on, you might find it informative to check the dates of publication of the various references cited–many concerns/warnings were published early in the pandemic or even pre-pandemic. Yet, although in hindsight these concerns and warnings have now been shown to have had some validity, at the time, they often went unheard or were dismissed.

We appreciate that the number of claims and counter-claims made by different groups throughout the pandemic was frequently overwhelming. Also, we want to emphasise the multidisciplinary nature of the scientific debates over various COVID-19 response measures. Experts from one relevant discipline often had expertise that could have improved the assessments of their counterparts in other relevant disciplines. Yet, the flow of knowledge between experts in different disciplines was limited.

Meanwhile, the relevant policymakers were essentially relying on a small subset of the experts, e.g., the members of various scientific advisory groups used by governments. This further restricted the flow of knowledge reaching the decision-makers who ultimately implemented policy responses.

For these reasons, decision-makers were making “science-based” decisions based on a limited subset of the scientific knowledge available at the time. Even as scientific knowledge accumulated during the pandemic, the flow of this knowledge to the decision-makers was similarly restricted.

In that sense, perhaps we can understand retrospectively why so much of the science that was followed by policymakers was later found to have been contradicted by other scientific research. Yet, regardless of why the various policies were implemented, it is important to investigate what lessons can be learned for future public health policies from how the COVID-19 pandemic was managed.

Therefore, in this article, we highlight what we believe were major problems in four main aspects of the management of the COVID-19 pandemic:

1. The over-reliance on COVID-19 models without adequate empirical evaluation
2. Insufficient critical evaluation of the non-pharmaceutical interventions (NPIs)
3. The inconsistent evaluation of different proposed pharmaceutical interventions (PIs)
4. The inadvertent dismissal of valid scientific perspectives as “misinformation”

The authorship of this article includes researchers from many disciplines–including immunologists, epidemiologists, virologists, public-health practitioners, pathologists, medical professionals, data analysts, economists, research methodologists, psychologists, medical doctors and social scientists. All of us have different perspectives on several of the topics we will discuss (e.g., the relative effectiveness of certain repurposed drug protocols or how useful the current mathematical models are for COVID-19) as well as different expertise. We believe that this multi-disciplinary collaboration between researchers with different perspectives provides us with a more holistic assessment.

On the other hand, we recognize that other researchers disagree with us–if our opinions were already universally shared and understood, then we would not need to write this article. Therefore, in what follows, the reader should remember that we are presenting our scientific opinions and we do not claim to speak for the entire medical or scientific community.

Our analysis looks collectively at global responses that were common to multiple countries, although when appropriate we will consider the cases of individual countries or regions that took a markedly different approach from other countries. For instance, the NPIs officially adopted by Sweden were widely recognised as being different from neighbouring countries [31–33] and the Chinese COVID-19 vaccines did not use the new mRNA/DNA technology that were used by many countries. This means that our analysis is a “big picture” overview of public-health responses that were implemented Globally, but the exact implementation of these general responses often varied in the specifics. For example, many countries implemented some forms of “proof-of-vaccination” policies, but the exact policies often differed between countries, e.g., see table 1 in Bardosh et al. [16]. Future research might delve into the details of the subtle differences arising from exactly how each of these responses was implemented in individual countries.

## Policy Lessons to Be Learned From the COVID-19 Pandemic

### Problem 1: The Over-reliance on COVID-19 Models Without Adequate Empirical Evaluation

“If you put tomfoolery into a computer, nothing comes out but tomfoolery. But this tomfoolery, having passed through a very expensive machine, is somehow ennobled and no one dares criticize it.” – Pierre Gallois (1911-2010) [38]

From the beginning, mathematical epidemiological models provided the key basis and rationale for most government responses to the pandemic [6, 39–48].

1. The initial abandonment of existing pandemic plans [49] and rapid replacement with a new *ad hoc* set of unprecedented (and largely untested) non-pharmaceutical interventions [7, 12, 50] seems to have been entirely based on deep concerns over alarming model projections provided to governments [43, 47] that predicted that millions of deaths would occur unless major NPIs were urgently implemented [6].
2. During the pandemic, decisions on whether to increase or decrease the stringency of NPIs were often heavily influenced by what model scenarios projected would occur if NPI stringency was reduced [39–42, 46, 51, 52].
3. The public health rationale for implementing population-wide vaccination programmes (as opposed to offering voluntary vaccination to individuals or specific at-risk segments of the population) was based on model scenarios of what would occur if NPI stringency were reduced before the estimated “herd immunity threshold” had been surpassed through mass vaccination [6, 44, 52–54].
4. Assessments of the effectiveness of both NPIs [7, 10, 12, 55–58] and vaccination programmes [59, 60] were mostly based on comparisons of what had occurred to “counterfactual scenarios” of what the models expected should have occurred in the absence of these policies.

We believe mathematical and computer models can often be very useful epidemiological tools and that modelling scenarios can be especially useful in the early stages of an epidemic [61, 62]. However, we are concerned at the *over-reliance* that was placed on modelling results for COVID-19 policies [39–42, 46, 51].

We also found it problematic that some models that provided a range of plausible scenarios and/or provided less alarming scenarios [51, 63–68] were apparently given less weight [69–72] than the more alarming projections [43, 47, 48].

Our biggest concern, however, is the absence of mechanisms by which the reliability of the models being used could be continually evaluated. As Box (1979) explained, “all models are wrong, but some are useful” [73]. However, unless the models are continually tested against reality, it is hard to identify the useful ones.

In this section, we will explain these concerns in detail.

#### Unsuitability of the 1920s SIR Mathematical Model Framework for COVID-19

In the early 20th century, Kermack and McKendrick (1927) developed a useful mathematical model for describing the progression of an epidemic, called the “Susceptible/Infected/Recovered (SIR)” model [74]. This model is a set of mathematical equations (differential equations) that compartmentalises the population into susceptible (S), infected (I), and then recovered (R) or dead. It offered an explanation as to how an epidemic of a highly infectious disease could end before everybody in the community had been infected [74].

Although the original SIR model was proposed in 1927 in the pre-computing era, it is also very amenable to being solved by more modern analytical methods [45, 61, 62]. It also could easily be adapted to include additional compartments such as an exposed (E) population that was not yet infectious, leading to the related SEIR model. Hence, the SIR framework, or its derivatives, still dominate the field of epidemic modelling. Indeed, most of the COVID-19 models published during the pandemic used some implementation of this SIR/SEIR framework [75] including those used to advise governments [39–46].

Some COVID-19 models also used computationally expensive agent-based models based on the SIR/SEIR framework, e.g., UK and USA [6, 43]; or Austria [44], but most studies used the simpler population-averaged models, e.g., USA [76]; Canada [77]; Ireland [45]; Spain and Italy [78].

**FIGURE 1:**
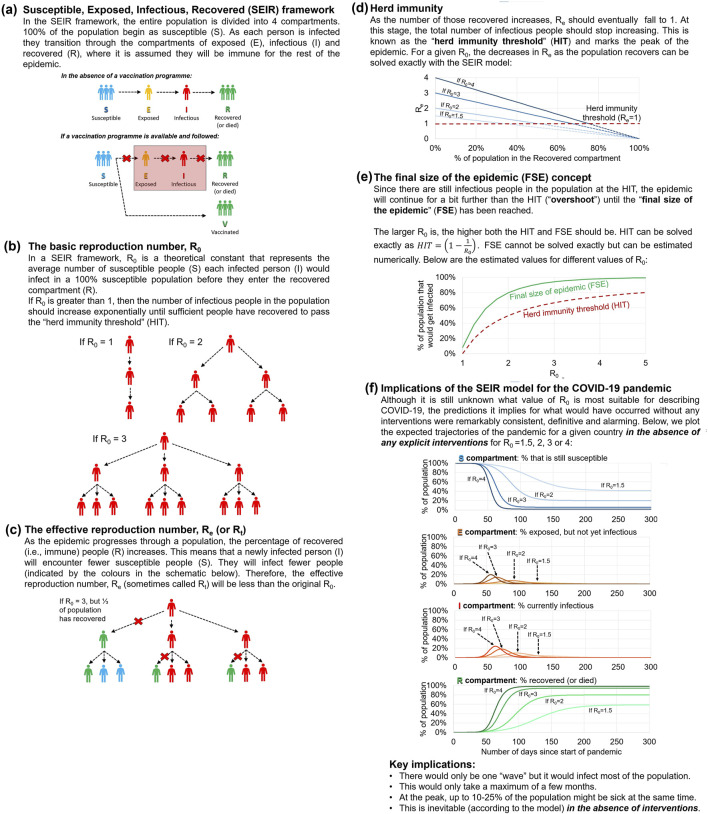
Summary of the implications of applying the Susceptible/Exposed/Infectious/Recovered framework to the COVID-19 pandemic. The Susceptible/Exposed/Infectious/Recovered model used for estimating population averages in the progression of a pandemic **(a)**, the basic reproduction number R_0_ **(b)**, the effective reproduction number R_e_ **(c)**, herd immunity **(d)**. The Susceptible/Exposed/Infectious/Recovered model used for estimating the final size of epidemic values **(e)** and the projections for the first 300 days **(f)** in the figure were taken from the model developed for the Irish Epidemiological Modelling Advisory Group by Gleeson et al. 2022 (downloaded from https://github.com/obrienjoey/ireland_covid_modelling; Last accessed 24/05/2022) [45].

In Figure 1, we explain the main features of the basic SEIR framework and demonstrate how it led to alarming initial projections [39–46, 76–78] when applied to the COVID-19 pandemic:

a. The default SIR/SEIR framework assumes that 100% of the population starts in the susceptible compartment but as the epidemic progresses everybody exposed to the virus will pass through each of the compartments until they have recovered (or die): S→E→I→R.
b. A key parameter for the model is the “basic reproduction number”, *R*_*0*_, a theoretical constant representing the average number of susceptible people each infected person would infect in a 100% susceptible population. If *R*_*0*_ >1.0, then the number of infected people will begin to increase exponentially over time; and the higher *R*_*0*_, the faster that growth rate.
c. However, the model predicts that as more and more people recover, each newly infected person will encounter less still-susceptible people on average. Hence, the “effective reproduction number”, *R*_*e*_ (sometimes *R*_*t*_), decreases over time.
d. When *R*_*e*_ decreases to 1, then the number of infectious people in the population stops increasing, marking the peak of the epidemic. This is called the “herd immunity threshold” (HIT).
e. The model predicts that the epidemic will still continue, albeit at a slower and slower rate, until the “final size of the epidemic” (FSE) has been reached.

Even now, it is still unclear what is the average *R*_*0*_ of COVID-19 [79, 80]. However, many of the early estimates suggested values in the range 2–6. Therefore, as can be seen from Figure 1F, the implications of the model projections were highly alarming. They projected that–in the absence of NPIs or a population-wide vaccination programme–there would be a major pandemic wave that would only take a few months to infect more than 70%–80% of the population. And at its peak, up to 10%–25% of the population would be sick at the same time–overwhelming health services, as well as devastating society.

When we are familiar with the implications of this model, we can understand why so many modellers were alarmed [39–46, 76–78] and why policymakers advised by these modellers could easily have shared this alarm. However, while the original SIR model remains a powerful useful epidemiological tool [61, 62], especially for highly infectious diseases that are predominantly spread through close contact, e.g., measles, there are multiple reasons why these simplistic projections were unrealistic for modelling the COVID-19 pandemic [39–42, 46, 51, 61, 63–66, 81].

##### Neglect of the Seasonality of Coronaviruses by Most of the Models

Most of the models did not consider the possibility of seasonality [6], e.g., the raw output of SEIR models is typically in “number of days since outbreak,” with a few exceptions [65, 82–85]. Hence, the model would predict an identical timeline for the pandemic, regardless of whether the outbreak was modelled as beginning in January or July.

The exact factors that contribute to the seasonality of respiratory viruses including influenza and coronaviruses are the subject of ongoing research and debate. For those interested in “why there is seasonality”, we recommend the review by Moriyama et al. [86] as a good starting point. We also note that the seasonality of individual viruses varies between genera, e.g., compare the different seasonal peaks of alpha-coronaviruses and beta-coronaviruses in Dyrdak et al. [87]. However, for this discussion, the key point is that the default SIR framework does not consider the possibility of a seasonal component.

Although the beginning of the COVID-19 pandemic was somewhat unseasonal (beginning in Northern Hemisphere spring) and cases were identified in summer periods, it is now apparent that there was a significant seasonal component to the dynamic of the pandemic (especially after accounting for changes in testing capacity). Other human coronaviruses in the same family as SARS-CoV-2 exhibit a strong seasonality in their incidence–reaching a sharp peak in mid-winter and having a very low incidence during the summer [82, 85–87]. Therefore, several studies suggested early on that this seasonality in coronavirus incidence might also influence the pandemic dynamics [82, 85–91]. Others disagreed and argued that SARS-CoV-2 should not be treated like other “seasonal” infections, because seasonality could not be (solely) used to characterise the early pandemic progression, e.g., outbreaks during summer and spring periods [4, 92–95]. Nonetheless, while these points rule out seasonality as the sole factor, most mid-to-high-latitude countries experienced major SARS-CoV-2 waves during the winter months and marked reductions during summer months, suggesting that seasonality is at least a *contributing* factor [88, 96–99].

In principle, the SIR model framework could be adapted to account for this seasonal component [65, 82–85]. However, with some exceptions [65, 82–85] this was generally not done. In some cases, this might have been an explicit decision, based on studies that insisted that COVID-19 was “not seasonal” [4, 92–95], but typically the studies describing these models [6, 44, 45, 76–78] did not even discuss the possibility of a seasonal component.

The neglect of seasonal factors was particularly noteworthy for the highly influential [43, 47, 48] Imperial College London model [6]. This model for the UK and USA had been repurposed [6] from an earlier model of a hypothetical influenza epidemic from Thailand [100]. In the Supplementary Material of that study, the authors had explicitly justified neglecting seasonality in this area because, while seasonality might explain waves in temperate climates, its effects were still unclear in tropical areas [100]. However, the template for that Thailand study was later reparametrized for an influenza model study in the USA/UK, which in turn became the basis for the COVID-19 model in Report 9, yet the authors neglected to reconsider the question of seasonality when doing so [6].

##### Misleading Projections of “One Wave” in the Absence of Interventions

A related shortcoming of the SEIR model is that it predicts that only one wave of the epidemic would occur in the absence of any non-pharmaceutical or pharmaceutical intervention–see Figure 1. This underlying assumption represents such a striking feature of the SEIR framework that researchers often prematurely interpret the presence of multiple waves of an epidemic as proof that interventions had temporarily flattened the epidemic curve. For example, in their analysis of the 1918 influenza pandemic in U.S. cities, Bootsma and Ferguson seem to use the existence of multiple waves and the fact that these waves were often lower in magnitude than that predicted by their SEIR model as conclusive evidence of the relative effectiveness of the different NPIs implemented by each city [101].

Therefore, modellers prematurely concluded that any deviations from the projected single large wave *must* be due to the government interventions, i.e., initially the NPIs [7, 10, 12, 55–57, 102], and later the vaccination programmes [59]. Hence, all declines in the incidence of the virus were automatically attributed to the interventions [7, 10, 12, 55–57, 59, 102], even if they had arisen from the seasonality of coronaviruses [88, 96–99] or the natural dynamics of epidemic waves [68, 103, 104] or the wave had already peaked before the interventions had been implemented [103–105].

##### Failing to Account for Heterogeneity in Susceptibility and Contacts

For simplicity, the original SIR model explicitly assumed that every person in the population will meet every other person with equal probability, like atoms in a mixture of gases and that they are equally likely to be infected [61]. Therefore, everybody is effectively treated as identical, regardless of age, gender, occupation, location, immune status, etc. The only difference between individuals is what compartment they are in at a given point in the epidemic.

While this approximation may apply well to a small community, it does not apply so well to a whole country, let alone the entire global population. People usually encounter the same family members, work colleagues and neighbours every day [61]. In addition, social mixing and disease transmission vary with age, because younger people tend to be more active than older people [106]. On the other hand, infection is likely to reduce the number of contacts an individual has, because people who become ill with an infectious disease are more likely to spend time in bed and out of circulation [61]. Many people will also be familiar with the concept of super-spreader events, where infectious transmission could theoretically be accelerated in large gatherings [107].

The more computationally expensive “agent-based” models partially addressed this concern by allowing for a more nuanced geographical analysis in terms of contacts than the more common population-averaged models [45, 76–78], e.g., distinguishing between rural and urban areas [100] and different behaviour between age groups [6, 44]. However, often these models had been calibrated to replicate the overall results of the population-based models [100]. Moreover, this only accounted for a small proportion of the person-to-person variability in transmission [108] and it did not allow for any heterogeneity in susceptibility, e.g., due to cross-immunity to other viruses [109].

Multiple studies have shown that models using a single average *R*_*0*_ to describe the average number of people infected by each person (rates of transmission) and the chances that an exposed individual will become infected (susceptibility) can be unrealistic [65, 66, 106, 107]. In contrast, models that allow for heterogeneity in either susceptibility to infection or transmissibility of infection can dramatically reduce both the expected herd immunity threshold (Figure 1D) and the final size of the epidemic (FSE) (Figure 1E) for a given wave [65, 66, 106, 107].

Models that account for this heterogeneity do not preclude the possibility of subsequent waves as the virus evolves [68]. However, they show that the key alarming prediction of the standard homogeneous SEIR model described in Figure 1, that the vast majority of the population would have become infected within a few months in a single wave without interventions, was flawed from the outset.

#### Inconsistencies in the COVID-19 Statistics Used to Calibrate the Models

Separate problems with how the models were calibrated and evaluated arose due to the inconsistencies in how the incidence of COVID-19 in the community was measured over time. Most models were fitted using either COVID-19 cases or COVID-19 deaths. However, the methods by which both of these metrics were measured changed throughout the pandemic [110–117].

In terms of “COVID-19 cases,” the proportion of the population tested, the priority of testing and the case definitions for COVID-19 changed dramatically over the course of the pandemic [110–116]. In the beginning of the pandemic, RT-PCR tests to reliably identify SARS-CoV-2 were still being developed [118] and testing capacity was limited. Hence, testing was often prioritized for patients with the most severe symptoms or healthcare workers (to reduce nosocomial spread in hospitals) [111, 119]. The case definition was also initially stricter and based more on symptoms than test results [113]. Therefore, the identified “cases” were initially skewed by the “clinical iceberg” effect [119, 120], i.e., many of those who were infected may have gone undetected because they did not present to a doctor for diagnosis and treatment.

This led to two major biases in the modelling of the early waves. First, the true number of infections during the first wave was substantially underestimated [111, 113–116]. It also meant that the early estimates of the infection fatality rate (IFR) and hospitalization risks were too high [1, 63, 111, 112, 121], since most of the infections with mild or even no symptoms would have remained unidentified [67].

As testing capacity increased (and also demand temporarily lessened) after the first wave, the case definitions were loosened [113] and testing priorities expanded. Hence, since mid-2020, the more number of cases identified was partially a function of testing capacity. The more tests undertaken, the more cases could be identified. This does *not* mean that the number of cases was merely a function of testing capacity. The number of tests carried out was a function of *both* the supply of tests *and* the demand for tests [122–124]. However, it meant that case numbers were an unreliable metric for studying the course of the pandemic.

Additional problems in the use of “case numbers” arose because COVID-19 testing laboratories often used very high cycle thresholds (Ct) of 40 cycles or higher in the RT-PCR test to reduce the possibility of giving a false negative to a sample from a person at the early stages of infection, i.e., when the viral load was still very low. This was useful in terms of containment strategies because the infectious period for COVID-19 seems to begin before or at the time of symptom onset [125], i.e., before the infected person realises they are sick.

The problem was that “case numbers” conflated both high-Ct and low-Ct positive specimens as identical, meaning that many of the identified “cases” were neither infectious nor symptomatic [126–130]. Hence, since mid-2020, many of the cases might have been completely asymptomatic and non-infectious, yet still treated identically to symptomatic and infectious cases [127, 128]. Some solutions might have been to either (a) include the Ct for a positive result [130]; (b) revert to a case definition that also required symptoms [113]; (c) provisionally treat a high Ct “positive” as possibly being pre-symptomatic (and advising quarantining), but following up with the person before confirming them as a “case”.

An alternative metric sometimes used for evaluating the pandemic progression was the number of COVID-19 deaths [117]. However, this metric also was surprisingly inconsistent, because some patients who had tested positive for COVID-19 but died of other causes were still counted as a “COVID-19 death”. For example, in the UK, deaths were recorded for people who died up to 28 days of having a positive COVID test or if COVID-19 was entered on their death certificate [117]. Therefore, “COVID-19 death” statistics combined together “deaths *from* COVID-19,” “deaths where COVID-19 was a contributing factor” and “deaths *with* COVID-19,” i.e., patients whose death had nothing to do with COVID-19 but who had coincidentally tested positive for COVID-19. This conflation of different causes of deaths into “COVID-19 deaths” has made it challenging to use these mortality statistics as a reliable metric for tracking the pandemic.

Meanwhile, testing of all hospital patients rapidly became routine to control nosocomial infections. This was understandable from a healthcare perspective, but it often led to confusion over the pandemic progression, because hospital patients who tested positive for COVID-19 while in hospital were typically counted among the “hospitalized *with* COVID” statistics even if the reason for hospitalisation had nothing to do with COVID-19 infection [131–133].

As well as leading to uncertainties in (a) the severity of COVID-19 morbidity and mortality risks, and (b) the relative magnitudes of the different waves of the pandemic over time, the above inconsistencies also would have introduced real-time errors into the data inputted into the models used for advising governments on how the pandemic was progressing in each country. Therefore, there are multiple reasons for strengthening the surveillance of infectious diseases.

#### Insufficient Efforts to Critically Evaluate the Reliability of Model Projections

A major concern with the over-reliance of policymakers on model scenarios is the lack of mechanisms implemented to try and assess the accuracy (or otherwise) of the models as the pandemic progressed [39–42, 46, 51]. This was especially necessary given that some models provided a range of plausible scenarios and/or provided less alarming scenarios [51, 63–68]. Hence, the most alarming scenarios were not necessarily the most plausible, yet the less alarming scenarios were apparently given less weight [69–72] than the more alarming ones [43, 47, 48, 134, 135].

Policies were determined based on what models projected would happen in the absence of those policies, i.e., essentially the dramatic scenarios outlined in Figure 1. However, typically, modellers did *not* attempt to model what would occur with those policies. Hence, once the policies were implemented, the model scenarios could never be tested by either the modellers or the policymakers, i.e., they were “non-falsifiable” [136].

This put policymakers in an unfortunate position. They were warned by modellers that if they did not implement major unprecedented policies, the consequences would be dire. Yet, once they implemented the policies, they were unable to assess how realistic those warnings had been. Nor had the modellers any feedback to establish whether they needed to modify their models going forward.

Meanwhile, most of the studies that retrospectively evaluated COVID-19 policies as relatively successful [7, 10, 12, 55–59] in reducing the spread of COVID-19 did so using so-called “counterfactual scenarios”. That is, researchers would compare the observed COVID-19 statistics following the introduction of the policies to the modelled scenarios of what might have happened in the absence of the policies. This comparison of an observed reality to a hypothetical “counterfactual scenario” was used to claim both NPIs [7, 10, 12, 55–58] and vaccination programmes [59, 60] were effective.

However, as we will discuss in the next sections, assessments that were *not* solely based on counterfactual scenarios often found that the progression of the pandemic was largely independent of government measures [64, 81, 96, 103, 105, 137–145].

Indeed, in a few rare cases where the policies recommended by the model scenarios were not implemented, thereby allowing a comparison of the model scenarios to the observed reality, the model scenarios were widely wrong [134, 135].

For instance, Sweden was somewhat unique in choosing to not adopt the wide range of NPIs implemented by neighbouring countries [31–33]. While many in Sweden may still have voluntarily modified their behaviour [32, 141, 142], these voluntary measures were widely regarded as less restrictive than mandated NPIs [31–33]. It therefore offers a rare counter-example for evaluating the effects of the NPIs implemented by neighbouring countries. Using Imperial College London’s own model, researchers estimated 34,895 first-wave deaths in Sweden under “social distancing of the whole population” — the most stringent measure short of full lockdown, and arguably the closest to the measures actually implemented in Sweden [134, 142, 146]. By the end of July 2020, the actual number of deaths reported in Sweden was 5,741 [31].

More broadly, the countries that implemented the least stringent NPIs did *not* generally experience more COVID-19 deaths than the countries with the most stringent NPIs [145, 147]. This is the opposite of what the models had predicted.

Lastly, after repeatedly following the model-based recommendations to implement strict NPIs for most of the pandemic, in December 2021, the UK government finally decided to overrule the model-based recommendations that were calling for a (fourth) lockdown in December 2021. On that occasion, the model predictions of deaths exceeded the actual numbers by a factor of 20 [70].

### Problem 2: Insufficient Critical Evaluation of the Non-pharmaceutical Interventions (NPIs)

“…there is always a well-known solution to every human problem—neat, plausible, and wrong.” – Henry L. Mencken, Prejudices: Second Series, p 155 (1920) [148]

Non pharmaceutical interventions (NPIs) are non-medical measures used to slow transmission of the virus and contain the pandemic. These might include bans on mass gatherings, travel restrictions, lockdowns, mask mandates, school closures and social distancing [7, 12]. Many of these measures were cloned by neighbouring governments, perhaps out of peer pressure [42]. A good review of the NPIs applied during the COVID-19 pandemic can be found in Inglesby et al. [50].

As we saw above, alarmed by model scenarios, governments introduced an unprecedented array of extensive NPIs across the world in early 2020 across the world in early 2020. Although governments would often temporarily reduce the stringency of these measures between waves of the pandemic, e.g., during the summer months, many NPIs were held in place throughout the pandemic [9]. These NPIs had many unintended negative consequences [42, 147, 149–156].

Moreover, although multiple model-based studies concluded that the NPIs were temporarily controlling the pandemic [7, 10, 12, 55–57], many studies found that the pandemic progression continued largely independently of the NPIs [64, 81, 96, 103, 105, 137–145]. Yet, NPIs almost exclusively comprised the bulk of COVID-19 policies until the COVID-19 vaccine programmes [7, 102], and remained a major part of COVID-19 policies throughout the pandemic [9].

We recognise that:

(a) The model scenarios presented to governments of what might unfold in the absence of NPIs were indeed highly alarming [39–46, 76–78].
(b) Therefore, if these model scenarios had been accurate, then attempts to prevent or minimise the modelled scenarios from unfolding should have been very high in terms of public health priorities, and the NPIs were such an attempt [7, 102].
(c) Several high-profile, counterfactual, scenario-based studies concluded that the NPIs were effective [7, 10, 12, 55–57] – on the basis that such scenarios had not unfolded when NPIs were implemented.

However, we believe that this NPI-driven strategy was seriously flawed for multiple reasons, e.g.,

1. Discussion of alternative strategies [32, 33, 49, 54, 137, 149, 150, 157–159] was prematurely dismissed without serious consideration [32, 33, 102].
2. By now, multiple studies have found the NPIs to have been remarkably ineffective [64, 81, 96, 103, 105, 137–145], yet there was a striking failure by policymakers to critically evaluate the effectiveness of NPIs throughout the pandemic.
3. The NPIs led to many unintended harmful consequences for not just public health, but also society and the economy [42, 147, 149–156].

We will elaborate on these points in the following subsections.

#### Dismissal of Alternative Strategies Without Serious Consideration

Before 2020, pandemic preparedness plans had been carefully prepared and they advised against most of the NPIs [49] that ended up being implemented. In particular, a systematic WHO review in autumn 2019 concluded that the evidence for the effectiveness of most NPIs was limited [49]. Additionally, the limited data in their favour was mostly based on either observational or modelling studies [49]. It was also predicted that NPIs might also have considerable harms [49]. These predictions were later confirmed at the end of the COVID-19 pandemic by a UKHSA evidence review of studies conducted in the UK [160]. Yet these pandemic preparedness plans that had been developed in advance were apparently abruptly abandoned with little discussion [161, 162].

Even during the early months of the pandemic, the WHO Strategic and Technical Advisory Group for Infectious Hazards (STAG-IH) recommended a range of measures for countries to prepare and respond to the pandemic that did *not* involve most of the NPIs that were ultimately used [163]. So, while the implementation of an unprecedented array of NPIs was frequently promoted as “following the science” [11, 48, 164–166], the reality was that most of the science that had been available at the time was hastily replaced on the basis of the alarming model projections [39–46, 76–78] described earlier.

During the pandemic, while the NPIs were in play, many researchers called on the community to reconsider this strategy and proposed alternative approaches to managing the pandemic going forward [54, 137, 149, 150, 157–159, 167, 168], yet these calls were either ignored or dismissed without adequate consideration [102, 168–173]. For instance, when an eminent epidemiologist wrote a generally favourable assessment in May 2020 of how Sweden had fared without the more stringent NPIs of its neighbouring countries [167] it led to six separate critiques disputing this assessment [102, 168–173]. Although Sweden did experience a relatively severe first wave due to a high death toll in elderly care services [174, 175] ultimately, it did *not* experience a noticeably more severe pandemic than its neighbours over the long-term [31–33, 96, 142].

In late 2020, three prominent epidemiologists wrote the Great Barrington Declaration (https://gbdeclaration.org/), advocating for a more “focused protection” strategy that prioritised the most vulnerable to COVID-19, particularly the elderly [176], rather than the diffuse strategies of population-wide NPIs that they were “gravely concerned” were leading to “damaging physical and mental health impacts”. At the time of writing, the declaration has had 941,261 signatories including 16,176 medical and public-health scientists and 47,839 medical practitioners. Yet, rather than leading to a public discussion over whether alternative strategies could be adopted, the declaration was immediately dismissed as allegedly promoting “*potentially dangerous fallacies”* [102]. Behind the scenes, the then-director of the National Institutes of Health apparently tried to organise “*a quick and devastating published take down of its premises*” because it “*seems to be getting a lot of attention*” [177].

We appreciate that governments were trying to respond quickly to the modelling advice provided to them. However, we believe that if governments had taken seriously existing pandemic strategies [49, 163] and/or sought feedback from researchers with multiple perspectives [54, 137, 147, 149, 150, 157–159, 167, 168, 178], they could have formed more well-rounded, evidence-based strategies than they did.

#### Failure to Adequately Re-Evaluate the Effectiveness of NPIs as the Pandemic Progressed

Given the speed of the adoption and implementation of NPIs, it is conceivable that governments neglected the proper critical appraisal of their effectiveness. This is perhaps reflected in early studies which suggested benefits of NPIs throughout 2020 and much of 2021, based on counterfactual scenarios [7, 12, 57, 179]. However, as discussed earlier, once the NPIs began being implemented, there seemed to be insufficient interest from policymakers in encouraging research to critically re-evaluate the effectiveness of the NPIs as more data accumulated. Additionally, governments likely felt a pressure to “do something” and to “keep up with” the more stringent NPIs implemented by their neighbours [112, 161, 162].

Governments appeared content to rely on model-based studies that concluded the NPIs must collectively be working whenever a pandemic wave was in decline [7, 10, 12, 55–58]. However, studies that looked closely at how the timing and magnitude of the NPIs in different countries compared to the dynamics of the pandemic in those countries failed to identify a clear or consistent influence of the NPIs [64, 81, 96, 103, 105, 137–145].

For instance, several studies found that COVID-19 waves had often peaked *before* the NPIs had been implemented [68, 103–105, 139]. This suggested that the rises and falls in viral incidence were largely independent of the stringency of NPIs. Indeed, retrospective analysis of sewage samples suggested that, in some countries at least, the disease may already have been present months before NPIs were even considered [180, 181]. Instead, several studies have suggested that the natural seasonality of human coronaviruses, reaching peaks in winter months and lows in summer months [82, 85–91], may have played a much greater role in the pandemic dynamics than the NPIs [81, 88, 91, 96–98].

Another challenge in assessing the effectiveness of NPIs was that, for much of the pandemic, governments typically simultaneously implemented a diverse array of completely different NPIs. This often made it very difficult to empirically isolate the relative effectiveness of any individual NPI. We note that many of the modellers, who argued that collectively the NPIs were effective, also shared this frustration–see the perspective review by Lison et al. [182]. We agree with Lison et al. that the simultaneous implementation of multiple different NPIs by multiple neighbouring countries severely hindered the scientific community’s ability to assess the relative effectiveness of individual measures [182].

We appreciate that many of the NPIs might *intuitively* feel like they should have been having a significant influence on the pandemic progression. However, scientific analysis often contradicts our intuitions.

Let us consider the wearing of masks, as a case study, since NPIs from mid-2020 often involved mask requirements [9, 145]. Mechanistic studies suggested that masks could potentially reduce the transmission of viral particles [180, 181]. However, meta-analyses of studies before the pandemic had failed to identify a statistically significant reduction in the spread of viral transmission for influenza [183]– even if some had identified a *non-statistically significant* possible effect [184]. Indeed, a randomized control trial in Denmark during early 2020 found that 2.1% (53/2,994) of the control group and 1.8% (42/3,030) in the mask-wearing group caught COVID-19 before the trial had to be discontinued as the government had introduced mask-wearing regulations [185]. Again, this difference was *not statistically significant*, and suggested that any effect was, at best, modest.

Including the Danish study, three randomized control trials (RCTs) have now assessed whether facemasks and respirators were effective in preventing COVID-19 transmission. In two of these studies, surgical or cloth masks were investigated [185, 186] and in the third study, N95 respirators were compared with medical masks in a multicentre, randomised study of health workers who had direct contact to patients with suspected or confirmed COVID-19 [187]. A Cochrane meta-analysis showed that, in conjunction with previous studies, these trials failed to demonstrate that masks significantly reduced viral transmission in the community or among healthcare workers [188]. In fact, high-quality data from randomized trials consistently failed to demonstrate a significant effect of masks on viral transmission, while evidence supporting the beneficial effect of masks was derived almost exclusively from lower-quality observational studies [189]. Additionally, a comparison of 35 European countries during the 2020–2021 winter failed to identify a statistically significant relationship between mask usage and COVID-19 outcomes [145].

#### Neglecting the Unintended Public-Health Consequences of NPIs

Public health involves much more than dealing with a pandemic [61, 147, 153, 190]. Yet, throughout the pandemic, many long-standing public-health policies and strategies from the pre-pandemic period [147, 153, 154, 156, 190] were abandoned or severely deprioritised to focus almost exclusively on just one public-health issue, i.e., minimising the spread of the SARS-CoV-2 virus [42, 147, 149–153, 155, 156]. Therefore, although NPIs were designed with a public-health goal of reducing the health burden of the pandemic [7, 102], public-health policies should have explicitly weighted the potential benefits of the proposed NPIs against their many unintended harmful consequences, not only on public health, but also on the wider society, economy, and natural environment [42, 147, 149–156].

While the NPIs often led to a mixture of beneficial as well as adverse consequences for different people and groups, the net consequences were often adverse, e.g., see ÓhAiseadha et al. [153] for an extensive review.

The harmful consequences of the NPIs included stress [191], adverse changes in diet, nutrition, body weight and obesity [192, 193], substance abuse [194, 195], tobacco smoking [196], emotional and mental health impacts [197, 198], impaired healthcare delivery [199], adverse economic, social and environmental impacts [200], interruptions of education [198, 199], reduced hospital attendance [155, 156, 201], reduced vaccine uptake [202, 203] and impaired care and health of the elderly [149, 204, 205]. The adverse impacts on lifestyle and population health were exacerbated by greater health inequalities according to age, gender, socioeconomic status, pre-existing health and location [153].

The harmful impacts of NPIs for body weight and obesity are particularly noteworthy in that there is some evidence for an association between overweight/obesity and an increased risk of long COVID [206, 207]. As mentioned in the Background, the possibility of long COVID was one of the concerns associated with the pandemic (after the risks of hospitalization or death) [3].

Therefore, we believe that most of the NPIs that were implemented during COVID-19 should be avoided, if possible, in future pandemics. If any of these measures are to be considered again, governments should ensure that this is in conjunction with rigorous and holistic cost-benefit analyses. Because of the far-reaching impacts of the NPIs, a proper impact analysis should also involve a more multi-disciplinary range of scholars from the social sciences and the humanities as well as public-health practitioners [147, 178].

### Problem 3: The Inconsistent Assessment of Potential Pharmaceutical Interventions (PIs)

“The physician must be able to tell the antecedents, know the present, and foretell the future – must mediate these things, and have two special objects in view with regard to disease, namely, to do good or to do no harm. The art consists in three things – the disease, the patient, and the physician. The physician is the servant of the art, and the patient must combat the disease along with the physician.” –Hippocrates, Of the Epidemics (c. 400 BCE) Book I, Section II [208]

While many of the COVID-19 policy responses focused on NPIs, these were also accompanied with key policies related to pharmaceutical interventions (PIs), especially from 2021 onwards when the COVID-19 vaccination population-wide programmes began. However, we have noticed that remarkably unscientific and inconsistent asymmetries arose in how different kinds of PIs were considered during the pandemic.

Specifically, research into the potential use of inexpensive repurposed drugs that had been identified as promising was actively discouraged rather than encouraged. Researchers who suggested that a particular protocol based on repurposed drugs might be safe and at least partially effective in the treatment or prevention of severe COVID-19 faced a continuous barrage of criticism and professional ridicule [209–212]. Protocols incorporating either hydroxychloroquine (HCQ) or ivermectin–two off-patent medications with well-understood safety profiles that have been widely used for multiple purposes for decades–were particularly penalised. People who wanted to use a repurposed drug protocol faced considerable difficulties, and physicians who wanted to offer such a treatment faced professional harm [211–213].

In contrast, research that promoted the use of a new class of vaccine technology (mRNA or DNA vaccines) was actively encouraged for COVID-19, and any research that questioned either the safety or effectiveness of these new types of vaccines was discouraged. Governments actively promoted the population-wide use of these particular vaccines–often explicitly using vaccine mandates and stigmatising the unvaccinated [16]. People who did not want to take these COVID-19 vaccines and doctors who tried to accommodate this request from patients faced unusual pressure [16, 210]. The experiences of people who suffered adverse reactions following their vaccination were actively disregarded and if they spoke publicly of their experiences, they were often ironically labelled as “anti-vax” [202, 214–218].

Meanwhile, many governments refused to accept WHO-approved COVID-19 vaccines using more conventional technology (inactivated virus-based vaccines) as being a valid alternative in terms of COVID-19 vaccine regulations [219]. Indeed, apparently the U.S. military engaged in online international propaganda campaigns to discredit the Chinese inactivated virus-based “Sinovac”/“Coronavac” [220].

Several of our co-authors have different scientific opinions on the relative safety or effectiveness of each of these PIs in treating or preventing COVID-19. Therefore, we recognise that the scientific literature on these topics is still evolving. Yet, we believe that enough information is already available to support the following positions:

1. The active discouragement of research into the identification and evaluation of potentially promising protocols involving cheap repurposed drugs was disturbing and disquieting–especially in those cases where the drugs under consideration had well-understood safety profiles.
2. The deliberate conflation of the new mRNA and DNA vaccine technology with traditional vaccines was misleading and led to a lack of genuinely informed consent among many who received or administered these COVID-19 vaccines.
3. The apparent effectiveness of the COVID-19 mRNA/DNA vaccines was incorrectly evaluated and seriously overestimated.
4. The apparent safety of the COVID-19 mRNA/DNA vaccines was also incorrectly evaluated and dangerously overestimated.
5. By late-2021, it was already self-evident that the COVID-19 vaccines were not suitable for reaching herd immunity and, for this reason, a continued *population-wide* vaccine programme (as opposed to offering voluntary vaccination for individuals) was no longer useful from a public health perspective.

In the following subsections, we will explain the reasons for each of these positions.

#### Active Discouragement of Research Into the Potential Use of Repurposed Drugs

From the start of the pandemic, physicians were told that no anti-coronaviral therapy existed and that healthcare workers were recommended to provide “supportive care only” and that other therapies should be avoided outside of randomized controlled trials [211–213]. The WHO explicitly prohibited the use of corticosteroids outside of clinical trials, until September 2, 2020 [221], when the WHO switched to recommending corticosteroids for severe and critical patients. Within hospital, supplemental oxygen therapy and potentially mechanical ventilation could be considered if necessary. However, patients that did not require hospitalization were typically told to rest at home without treatment, but to “return to hospital if they develop any worsening of illness” [222].

Given the explicit absence of *any* treatment options, it is not surprising that some physicians and researchers began looking at the possibility of developing potential protocols for treatment and/or prevention by repurposing promising drugs [223, 224] – preferably well-studied and affordable candidates with known safety profiles [209–213, 223–230]. What was surprising was the strongly hostile over-reaction from the medical community and health authorities whenever a potential protocol was identified as promising [210, 231, 232]. This was often accompanied by media campaigns to create the public impression that these recently-proposed protocols were not simply ineffective, but potentially dangerous (even if the protocol had only recently been proposed and it was based on widely used drugs), and that physicians that were considering these protocols were therefore behaving dangerously and recklessly [233]. A recurring theme used in media campaigns to discredit these drugs, that are so widely used which they have applications for both humans and animals, was to emphasize the veterinary usage, hence creating the false impression that the drugs might not be suitable for human usage [234, 235].

Probably the most high-profile potential protocols were those that included either hydroxychloroquine (HCQ), ivermectin or anti-inflammatory medications such as corticosteroids (dexamethasone) [209, 210, 213, 223, 224, 227–230]. These drugs had been widely used by the medical community before the pandemic and therefore their safety profiles were well understood, making them promising candidates for repurposing [223, 224]. Several promising protocols involving their use were suggested as a “short-term option for the early treatment of most symptomatic high-risk outpatients” [209, 226]. Different studies of HCQ have given conflicting results, with some studies finding no mortality benefits to the use of HCQ [236, 237] and others finding a statistically significant mortality benefit [238]. However, on the basis of the former, in June 2020, the US FDA rescinded its temporary emergency use authorisation for HCQ [239]. Physicians who attempted to provide patients with access to protocols involving HCQ–even as a “right to try” – were either stonewalled or faced professional censure [210–212, 231–233].

In contrast, even though the Solidarity Therapeutics Trial coordinated by the WHO found in October 2020 similarly negative results for both HCQ and remdesivir (a relatively expensive drug still under patent by Gilead Sciences) [237], the WHO still allows the use of remdesivir in certain circumstances [221].

Another proposed repurposed drug, ivermectin, had been in use for several decades as a safe, inexpensive antiparasitic drug [225]. Ivermectin already demonstrated antiviral activity against other RNA viruses including HIV, influenza A and SV40 (DNA virus) in lab tests by a host-directed nuclear import protein inhibitor (IMP) [229]. Again, different studies have given conflicting results on its efficacy [225, 228, 240–243]. However, the tested protocols have not shown any toxicity issues [225, 228, 240, 241]. Moreover, one double-blind, randomized clinical trial of ivermectin reported it to be a potentially safe and effective medication for COVID-19 patients with moderate disease [242] and another review and meta-analysis states that “ivermectin could reduce the risk of mechanical ventilation requirement and adverse events in patients with COVID-19, without increasing other risks. In the absence of a better alternative, clinicians could use it with caution” [243].

A detailed review on the relative effectiveness of the various protocols involving HCQ, ivermectin or any other cheap repurposed drug is beyond the scope of this article–indeed several of our co-authors have different views on these ongoing debates. So, in this current article, we are *not* necessarily drawing any definitive conclusions as to their effectiveness. Nor are we saying that treatments needed to be based on cheap drugs. Indeed, monoclonal antibodies were granted EUA in November 2020 by the FDA in the USA and are used to treat and also detect COVID-19 [244].

However, all of the co-authors are alarmed at the manner in which research into the potential use of protocols involving the use of widely-used cheap repurposed drug was not just discouraged but vilified. Even if none of these protocols had been effective, given the major worldwide usage and well-understood safety profiles of many of these drugs before the pandemic, open-minded exploration into their potential should have been welcomed [209, 210, 225], rather than attacked [210, 226, 231, 232]. Especially since most patients were not provided with any alternative treatment–other than, from 2021, hoping that the mRNA/DNA vaccines that we will discuss below would help.

#### The Conflation of New mRNA and DNA Vaccine Technology With Traditional Vaccines

For decades before the pandemic, major public campaigns to encourage various population-wide and individual vaccine programmes, coupled with the labelling of opposition to any vaccine as “unscientific” and “anti-vax”, had led to a common public perception that any vaccine that was offered to the public by health authorities should automatically be trusted as being “safe” and relatively “effective” and based on well-established science [245–247].

This meant that when the various “mRNA vaccines” and “DNA vaccines” introduced in late 2020 were described as “vaccines”, many people–including high-profile doctors and scientists that took and promoted the mRNA/DNA vaccines–did not realise until much later that these new vaccine technologies were not the same as traditional forms of vaccination and had not previously been used for any public vaccination programmes before 2020 [218].

From a marketing perspective, this remarkable public trust in the term “vaccine” was very useful for developers of these new technologies (e.g., Moderna, AstraZeneca, Pfizer), who had been trying to develop an mRNA or DNA “vaccine” that would be commercially relevant for more than a decade [248, 249] It also seems to have contributed to many people (including researchers and health authorities) mistakenly conflating concerns over these specific vaccines with general “vaccine hesitancy” [250].

However, mRNA and DNA vaccines were very different from the conventional vaccines the public had been used to up to 2021. Traditional vaccination strategies use the whole or part of an inactivated or weakened infectious agent to stimulate the immune system into providing protection against further attack. In contrast, the theory behind these new mRNA/DNA vaccine technologies is to use DNA or mRNA to instruct human cells to produce part(s) of the viral protein which are subsequently displayed on the outside of the cell membrane. The idea is to mimic a part of the infective process of the virus. In theory, the human immune system should then recognise viral protein as non-self or foreign, try to destroy the cells that display it, and then create antibodies that can recognise it in future encounters [251].

Although the technology that these genetic vaccines were derived from promised much for the biotechnological industry back in the early 2000s, its regulatory approval was delayed for more than a decade due to significant adverse events. To overcome this hurdle, the genetic code of parts of the virus (that would act as a vaccine) was extensively modified so that it could bypass the human innate immune system [251] paving the way for repurposing this technology for the new vaccines. However, there are still concerns that this new type of vaccine technology was only approved by emergency use authorisation (EUA) via suspension of normal testing processes and review, rather than the more fitting regulatory approval as gene therapy [252].

**FIGURE 2:**
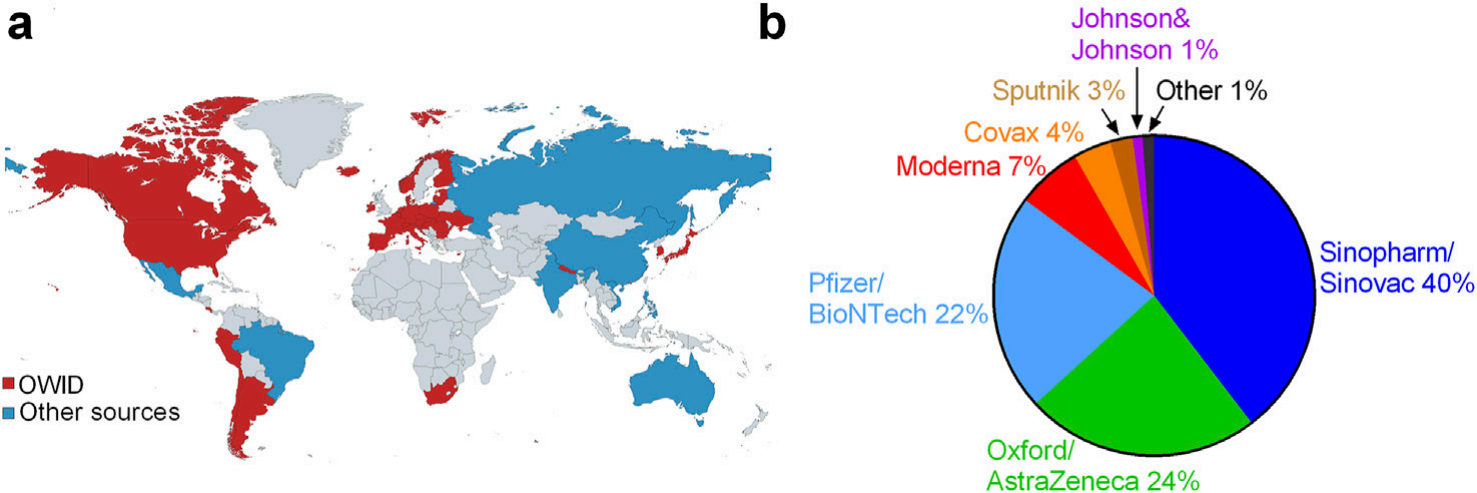
Global roll-out of COVID-19 vaccines: **(A)** the 50 countries where these vaccines have been rolled-out (and the data is available) with data from Our World In Data [254] (red), and approximated from webpages (listed in Supplementary Data File Excel S1) for Brazil, China, Mexico, Vietnam, Russia, Philippines, Australia and India (blue) and **(B)** global proportion of each vaccine by manufacturer (World, 2023).

However, COVID-19 vaccines were also developed and received WHO approval using more conventional processes especially in China and India, e.g., Sinopharm, Sinovac and Covaxin [34, 35, 253] and these were used in many countries, especially in the developing world–see Figure 2. In many countries, only the mRNA or DNA vaccines were accepted as valid in terms of COVID-19 vaccine regulations [219].

We believe that if the “mRNA/DNA vaccines” had been labelled using a terminology to better indicate (a) their novel (and relatively untested) genetic nature [252, 255–261] and (b) that they were not the traditional “vaccines” that people were familiar with, this would have allowed health authorities to better assess their suitability for public-health purposes and help the public make their decisions with informed consent.

#### Incorrect Evaluation and Serious Overestimation of the Effectiveness of the COVID-19 mRNA/DNA Vaccines

##### Initial Evaluation of Vaccine Efficacy

By early 2021, multiple pharmaceutical companies had announced COVID-19 vaccines that they declared were safe and efficacious at preventing symptomatic COVID-19 illness [13–15]. The initial trials of the COVID-19 vaccine were greeted with great enthusiasm, partially due to the promise of ending restrictive NPIs [21]. In particular, the Pfizer/BioNTech BNT162b2 mRNA COVID-19 vaccine (“Pfizer”) and Moderna mRNA-1273 SARS-CoV-2 vaccine (“Moderna”) were described as being 94%–95% efficacious in preventing symptomatic COVID-19, based on relative risk reduction (RRR) [13, 15]. Based on these trials, several of these COVID-19 vaccines were approved for full or emergency use authorization (EUA) – see Figure 2 for a breakdown of the worldwide distribution of the COVID-19 vaccines by the end of the WHO’s international public emergency (May 2023).

Although the clinical trials were not designed to evaluate if the vaccines had any influence on viral transmission [261–263], it was argued that if the vaccines prevented COVID-19, then this should reduce viral transmission in the community [264]. Coupled with the safety claim (which we will discuss later), this high RRR rate appears to have convinced public health authorities that it would be suitable for a population-wide vaccination programmes to pass the herd immunity threshold without much further illness (Figure 1A) and thereby prevent the unprecedented public-health catastrophes the models were still predicting would occur if the NPIs were removed [44, 52–54].

However, this optimistic justification in early 2021 for implementing population-wide vaccination programmes using these new mRNA/DNA vaccines would probably not have been as compelling if the corresponding results based on the absolute risk reduction (ARR) had been considered–see Table 1. This is because the high RRR values for these vaccines were based on a surprisingly small sample size, i.e., about 200 cases per vaccine trial or 1,222 cases out of 197,398 participants across all 7 trials [261, 265–267].

**TABLE 1:** Global vaccine trial data randomized studies. Cases defined as symptomatic with positive RT-PCR test. Relative risk reduction (RRR) and absolute risk reduction (ARR) (World, 2020).

Vaccine	Participants		Cases identified		Trial sites	Cases/site	RRR %	ARR %
	Vaccine	Control	Vaccine	Control				
Pfizer/BioNTech [15]	21,720	21,728	8	162	152	1.12	95.06	0.71
Moderna [13]	15,210	15,210	11	185	99	1.98	94.05	1.14
Oxford/AZ [14]	5,807	5,829	30	101	30	4.37	70.18	1.22
Sinopharm [34]	26,924	13,458	47	95	3	47.33	75.27	0.53
Sinovac [35]	6,646	3,568	9	32	24	1.71	84.90	0.76
Johnson & Johnson [36]	19,630	19,691	116	348	8	58	66.56	1.18
Sputnik V [37]	16,501	5,476	16	62	25	3.12	91.41	1.04

Hence, while the rates of 66%–95% relative risk reduction in Table 1 initially seem very impressive, when we realise that only 0.5%–1.2% of the participants in either the vaccine or control arms were identified as confirmed cases during the trials, the ARR results are much less impressive. These ARR results were reported alongside the RRR results and noted by several researchers at the time [261, 265–267]. However, they do not appear to have been considered in the initial justification for the population-wide vaccination programmes [261, 265–267].

Moreover, one of the reasons why only 0.5%–1.2% of trial participants in either arm were identified as cases during the trials seems to be that many participants with suspected but unconfirmed COVID-19 were not included in the RRR calculations. For example, for the Pfizer trials, while the RRR of 95% was based on 170 confirmed cases (of which 95% were in the placebo group), there also were *“1594 cases of suspected but unconfirmed COVID-19 in the vaccine group* vs. *1816 in the placebo group*” [263]. Hence, the number of participants with “suspected but unconfirmed COVID-19” was 20 times higher (3,410) than the 170 confirmed cases used for calculating RRR. Additionally, a further 371 participants (311 from vaccine group vs. 60 from placebo group) were excluded from efficacy analysis for “*important protocol deviations on or prior to 7 days after Dose 2*” [263]. It is still unclear how many of the 3,410 suspected but unconfirmed cases in the Pfizer trial would have been confirmed cases if adequately tested and how this would have affected the RRR. However, the fact that only 170 confirmed cases were identified out of the 43,448 Pfizer participants across 152 trial sites (1.12 cases/site–see Table 1) is surprising. This is extra concerning given the whistle-blower testimony from a former regional director of one of the Pfizer clinical trial sites indicating that inadvertent unblinding of the trial participants to the trial staff was potentially common in this study [268, 269]. This is because, if any staff involved in the procedures for testing suspected cases were unblinded, it could have potentially biased the results–even if unintentionally.

##### Changing Narrative About the Apparent Effectiveness of the COVID-19 Vaccines

Despite the concerns about the reliability of the clinical trial RRR efficacy values described above, by mid-2021, several observational studies had been published suggesting a vaccine effectiveness of more than 90% [270–273]. Hence, promoters of the COVID-19 vaccines still had studies they could cite to support their effectiveness.

However, many of these observational studies suffered from major flaws and were plagued by multiple confounding factors [26, 274–281] that undermined the reliability of these optimistic results [275].

These statistical biases included the miscategorisation bias; age bias; biases due to changes in the background infection rate during the study period [274, 275, 277–280], as well as the so-called “healthy vaccinee effect” [276]. These biases are often subtle, and in that sense, it is perhaps not surprising that many people (including co-authors of the observational studies) might have overlooked them. However, the biases are very important to consider because they often have the effect of boosting the *apparent* vaccine efficacy (VE). Indeed, they could even inadvertently make a hypothetical placebo (with no VE by definition) seem to have a very high VE [26, 274–281].

This problem has been illustrated succinctly by Fung et al. (2023) in which they demonstrate how three different biases common to multiple COVID-19 vaccine observational studies can each make a vaccine mistakenly seem to have a much greater VE than it does. Specifically, they showed how, using a similar study design to many of these observational studies, an apparent effectiveness as high as 50%–70% could in theory be calculated for a placebo treatment with a 0% actual effectiveness [275].

Many of these observational studies have been shown to be affected by at least one (and often several) of these biases. For instance, Neil et al. identified 38 observational studies that implied a high VE for one or more of the COVID-19 vaccines but had failed to account for the “miscategorisation bias” [279]. They concluded that, “Simulation demonstrates that this miscategorisation bias artificially boosts vaccine efficacy and infection rates even when a vaccine has zero or negative efficacy. Furthermore, simulation demonstrates that repeated boosters, given every few months, are needed to maintain this misleading impression of efficacy” [279].

Hence, we suggest that the apparently high VE results implied by individual observational studies should be treated cautiously until they have been shown to have adequately accounted for these statistical biases [26, 274–281].

At any rate, many countries had reached high levels of COVID-19 vaccination by mid-to-late 2021. However, despite the reported high RRR efficacy values, cases soon began to emerge amongst vaccinated persons (dubbed “breakthrough cases”) as well as the unvaccinated, accompanied by reports of decreased vaccine effectiveness [17–20, 22, 282]. It soon became apparent that vaccination was not preventing viral transmission either [283, 284].

This prompted an apparent shift in the rationale for taking the vaccine–it was argued that the vaccines could potentially prevent severe illness and hospitalisation [16, 18, 21, 22], although this had not been demonstrated by the randomised clinical trials [262, 274]. These new claims that the vaccines reduce the severity of symptoms, that at the time of writing the WHO asserts to be correct (https://www.who.int/news-room/fact-sheets/detail/coronavirus-disease-(covid-19), last accessed 22/04/2025) appear to be based solely on observational studies with similar design flaws to those mentioned above [26, 274–280].

Even for those groups with a low risk of hospitalization or death from COVID, e.g., younger groups, it has been suggested that vaccination might potentially reduce the risk of developing long COVID [285–287]. Some studies even suggested it might work as a potential treatment for long COVID [285, 286, 288]. Indeed, one of us, who was suffering from long COVID underwent COVID-19 vaccination because of this hypothesis, but in this case it did not help.

However, attempts to estimate whether COVID-19 vaccination might offer a protective effect against long-COVID remain inconclusive [285–289]. For instance, in a retrospective analysis of 19 million NHS records, yielding 55,465 documented cases of long COVID, in England (UK) up to January 2023, Henderson et al. (2024) concluded, “It is unclear what role vaccination had in the protection against long COVID, beyond reduced risk of any infection.” [289].

Meanwhile, others have noted that some adverse reactions suffered by some vaccinated people share many similarities to long-COVID symptoms [216, 218, 290], with some even referring to long-lasting COVID-19 vaccination adverse reactions as “long vax” [290].

Nonetheless, by comparing official COVID-19 death statistics to counterfactual scenario model projections of what might have occurred without vaccination, Watson *et al.* (2022) concluded (based on the assumption of 95% vaccine effectiveness) that COVID-19 vaccination had prevented 14.4 million COVID-19 deaths up to December 2021 [59]. A similar counterfactual scenario analysis concluded that the U.S. COVID-19 vaccination programme had prevented 18 million hospitalizations and 3 million deaths in the United States up to November 2022 [60]. Therefore, apparently on this revised narrative that the vaccines might reduce COVID-19 severity, the population-wide vaccination programmes were continued, and previously vaccinated people were encouraged to take additional, booster doses [16, 18, 19].

#### Incorrect Evaluation and Dangerous Overestimation of the Safety of the COVID-19 mRNA/DNA Vaccines

Reports of the phase 3 trial results for the various COVID-19 vaccines claimed that each vaccine was safe (as well as efficacious) [13–15, 34–37]. This was typically achieved by noting that the incidence of severe adverse events and/or fatalities were (a) rare and (b) similar in both the vaccinated group and the control group. On this basis, governments began population-wide vaccination programmes in early-2021.

The trials for evaluating safety of each vaccine were relatively short (often <2–3 months) and at the end of the trials, participants in the control groups were unblinded and offered the vaccine–thus preventing continued evaluation of the groups over longer periods. This is despite the fact that vaccines require a very high safety standard because they are administered to healthy individuals [249, 291] and because adverse events might not be confirmed until years after the roll-out [291]. The brevity of these safety trials was particularly concerning in light of the novel nature of the mRNA and DNA technology [257–261].

As the vaccination programmes progressed–and towards the end of 2021 as they switched to booster programmes involving third or subsequent doses–the narrative of what was “safe” seems to have progressively evolved, as the estimated incidence of concerning adverse events increased by orders of magnitude over time as we will discuss below.

Typically, the frequency of side effects associated with a medication are commonly annotated as follows:

• “Very rare” denotes a side effect that occurs in less than 1 in 10,000 people, i.e., <0.01%
• “Rare” is between 1 in 10,000 and 1 in 1,000, i.e., 0.01%–0.1%
• “Uncommon” is between 1 in 1,000 and 1 in 100, i.e., 0.1%–1%
• “Common” is between 1% and 10%
• “Very common” is more than 10%

In early 2021, concerns initially focused on “rare” cases of serious blood clotting events associated with two of the DNA vaccines–AstraZeneca and Johnson & Johnson–especially among women [292–294]. More than 30 of the first 222 suspected cases in Europe subsequently died [292].

In parallel, concerns over the mRNA vaccines initially focused on cardiovascular issues chiefly, myocarditis/pericarditis. Although early assessments based on passive adverse event reporting systems initially suggested these events were “very rare” [295], it soon became apparent that the rates were higher among younger men [296, 297] and increased substantially from the first to second dose [297–299].

In the United States in June 2021, the Advisory Committee on Immunization Practices (ACIP) debated the benefits/risks of continuing the programmes for young adults. They explicitly assumed that the vaccines were 95% effective “in preventing COVID-19 cases and hospitalization” [300]. Hence, they calculated that the benefits outweighed the risks [300]. As we saw above, such optimistic assumptions of vaccine effectiveness were soon abandoned.

Some studies conceded that the mRNA vaccines led to an increased risk of myocarditis and pericarditis, but argued that the risks were higher from COVID-19 infection [270]. It was later realised that such estimates were flawed, because the numbers of COVID-19 infections during the first waves had been severely underestimated and the risks from infection had been overestimated [301]. Studies that avoided this problem confirmed that the risks of myocarditis and pericarditis were much higher from vaccination than from infection. For example, between May and October 2021, 32 million people aged 12 to 50 were vaccinated with the mRNA vaccines in France and 3,225 members of that population developed myocarditis or pericarditis over the same period (i.e., 0.01%), but 97% of these cases were due to the vaccines, not infections [299].

By 2022, studies of vaccine-induced myocarditis/pericarditis that were stratified by sex, age, dose number and vaccine brand found much higher estimates of the incidence of this condition, at greater than 1.5 in 10,000 (“rare”) among young males after the second dose [301].

More recently, several high-sensitivity observational studies of vaccination programmes within individual institutions have indicated that cardiovascular manifestations after the second dose are “very common”, but usually “mild and temporary” [302, 303]. Participants with either abnormal ECGs or elevated cardiac biomarkers were “common” [302–304], but all patients in these studies had fully recovered by the end of the study [302–304]. These studies suggest that the incidence of mild and transient myocarditis and/or pericarditis is not “rare”, but rather “uncommon” [302–304].

As time has elapsed, new information has raised concerns about how accurately the safety data during the original clinical trials had been reported and interpreted. A reanalysis of the Pfizer and Moderna clinical trial data suggested that there were 0.10%–0.15% serious adverse reactions relative to the placebo arm [305], i.e., “uncommon” rather than “very rare” as originally claimed.

Meanwhile, an analysis of suspected adverse events following Pfizer vaccination in Denmark suggested that some vaccine batches had much higher rates than others [306]. That said, this study was controversial and disputed on multiple fronts [307–310]. So, while many of these criticisms have been responded to [306], the results should be treated cautiously. Nonetheless, subsequent studies have found similar findings for Sweden [311] and the Czech Republic [312], suggesting that this controversial finding may require further investigation. If the finding turns out to have some validity, the potential inconsistencies between batches could increase the difficulties in accurately estimating the risks of adverse reactions from these vaccines.

In November 2021, a former regional director of one of the Pfizer clinical trial sites provided whistle-blower testimony alleging serious concerns over data integrity and regulatory oversight in the sites she was working at [268, 269]. This included concerns that the adverse reactions of some trial participants were not being adequately followed up. Indeed, Brianne Dressen (AstraZeneca trial participant) and Maddie de Garay (Pfizer 12–15 years old trial participant) have both publicly spoken about how:

(a) Their severe adverse reactions were omitted from the papers describing the trial results.
(b) They experienced psychological manipulation (“medical gaslighting”) rather than medical support from the trial organisers.
(c) They were still struggling with severe vaccine injuries a year after the trials [215–217].

Only 1,131 participants had been in the vaccine arm of Maddie de Garay’s clinical trial [313], so her case accounted for 0.089% of vaccinated participants. Yet, neither of these two trial participants were mentioned in the studies describing the safety and efficacy of the relevant vaccines [14, 313].

As the estimates of the rates of adverse reactions increase over time, the range of possible adverse reactions confirmed to be associated with the mRNA and DNA vaccines also continue to increase: e.g., Guillain-Barré syndrome [314]; cerebral venous sinus thrombosis [314]; encephalomyelitis [314]; psychiatric adverse reactions [315] menstrual irregularities [316, 317] tinnitus [318] and shingles [257]. In some cases, at least, the vaccines have been linked to fatal outcomes [319, 320].

We also note that some studies have found instances of the genetic material of the vaccine or spike protein fragments remaining in circulation for months [257, 321–323]. For example, one study detected recombinant spike protein fragments in the blood of patients 187 days after vaccination [321]. Another noted the presence of mRNA concentrated in extracellular vesicles of human breast milk [322]. This is concerning as it was initially assumed that this genetic material and its protein product would be short-lived [249].

Rasmussen Reports have carried out a series of polls of the general public in the United States that have included questions on the COVID-19 vaccines. Below, we list a few recent examples, but see https://www.rasmussenreports.com/search?SearchText=covid for the most recent results:

• May 20–22, 2024 (N = 1,250): “Do you know someone personally who died from side effects of the COVID-19 vaccine?” 19% yes; 74% no; 7% not sure. [For comparison, the results for “Do you know someone personally who died from the COVID-19 virus?” were 42% yes; 53% no; 5% not sure.]
• March 27–29, 2023 (N = 1,078). “Has any member of your household died whose death you think may have been caused by side effects of COVID-19 vaccines?” 10% yes; 85% no; 5% not sure. [For comparison, the results for “Has any member of your household died from COVID-19?” were 11% yes; 86% no; 3% not sure.]
• November 30-December 1, 2022 (N = 1,000): “Do you believe you have experienced major side effects, minor side effects or no side effects from your COVID-19 vaccination?” 7% major side effects; 34% minor side effects; 56% no side effects; 4% not sure.

Obviously, such polls only capture personal non-expert opinion, but they indicate that (rightly or wrongly) a sizeable minority of the public personally believe that they or people they know suffered a severe adverse reaction from the COVID-19 vaccines.

Finally, there are still concerns over significant increases in excess mortality during the period associated with the vaccine roll-out compared to either the pre-vaccination period of the COVID-19 pandemic (2020) or pre-pandemic years [261, 324–326]. Indeed, some studies comparing all-cause mortalities between vaccinated and unvaccinated groups found slightly increased risks among vaccinated groups compared to the unvaccinated [327, 328] i.e., the opposite of what might be expected if the vaccines were reducing mortality risks. Although there are many problems in the attribution of excess deaths in any particular country, mainly due to the number of possible causes involved, and in identifying a reasonable and consistent measurement system, the relationships of the timings of increased mortality to vaccine roll-outs is an important “safety signal” [325], and the fact that “excess mortality has remained high in the Western World for three consecutive years, despite the implementation of containment measures and COVID-19 vaccines” [326] raises serious concerns. It also suggests that the vaccine adverse events reporting system (VAERS) is not operating as intended and that safety signals are being missed [329].

#### Continuation of Population-Wide COVID-19 Vaccine Programs After Late-2021

##### General Concerns Over Vaccine Mandates

The original justification for carrying out *population-wide* vaccination programmes was based on the explicit assumption that vaccinating the population past the theoretical “herd immunity threshold” would substantially reduce viral transmission [16]. Therefore many governments introduced a fairly coercive policy of vaccination, in many cases making it mandatory or at least “socially difficult” for people to make an informed choice [16]. The ethics of vaccine mandates has also been heavily debated in the media and scientific literature, with some pointing to their unethically coercive nature. [330]. We believe that these mandates breached the core ethical principles of informed consent and bodily autonomy.

Bardosh et al. point out that the inadvertent consequences of these policies exacerbated current health and socio-economic inequalities, increased social polarisation and lowered confidence in governmental and public-health institutions [16]. They also caused practical problems such as staff resignation [331].

Finally, we question whether it is ethical to mandate any medicine on any section of the population where there is a demonstrable net harm, e.g., young adults and children whose risk of severe COVID-19 is much lower than the risk of vaccine-injury from the mRNA vaccines [332–334].

##### Disappearance of the Rationale by Late 2021

In any case, as we discussed earlier, by late 2021, it was abundantly clear that the vaccines did not prevent COVID-19 infection [17–20, 22, 282]. It also was clear that vaccination did not prevent viral transmission either [283, 284]. Therefore, the public health basis for a population-wide vaccination programme (see Figure 1) no longer applied.

The WHO currently argues that the vaccines still “provide strong protection against serious illness, hospitalization and death” (285), based on observational studies suggesting that the vaccines might reduce the severity of illness [16, 18, 21, 22], although, as discussed earlier, the statistical reliability of these claims has been disputed [26, 274–278, 280]. Therefore, an argument could be made that individuals might choose to voluntarily continue a personal vaccination programme through boosters, especially those at a high risk of severe illness–e.g., elderly or severely obese [174, 335–337]. However, there was no longer a public-health argument for continuing a *population-wide* vaccination programme.

Moreover, the virus continued to evolve during the pandemic and by early 2022 it had evolved into a much milder, “omicron” variant, which appeared to be more transmissible but less virulent than earlier variants [338, 339]. Meanwhile, recovery from infection was shown to provide protective immunity for subsequent infection that was at least as effective as vaccination [340, 341], yet COVID-19-recovered patients were often encouraged to continue their vaccination programme. Therefore, the percentage of the population that was at risk of severe illness continued to decrease.

Finally, we discussed earlier how the confirmed risk of serious adverse reactions from the COVID-19 vaccines has increased over time [296, 301–305, 314]. This meant already by early 2022, for some demographics, e.g., young males, the confirmed risk of harm from the vaccines was orders of magnitude higher than their risk of developing severe COVID-19 [342, 343].

### Problem 4: The Inadvertent Dismissal of Valid Scientific Perspectives as ‘Misinformation’

“I would rather have questions that cannot be answered, than answers that cannot be questioned.” – Anonymous (sometimes attributed to Richard Feynman, 1918–1988).

One of the recurring themes of the public discourse around COVID-19 has been the importance of “following the science” [11, 48, 164–166]. However, throughout the pandemic, researchers could (and did) reach different scientific opinions on many aspects of the science related to COVID-19 and/or the handling of the pandemic. Moreover, these particular scientific opinions sometimes changed in light of additional information [25–27, 33, 41, 42, 48, 166, 232, 344–347].

Simultaneously, the public was inundated with often-changing narratives of what “the science says” [11, 48, 102, 164, 166, 346]. People who disagreed with any aspect of these narratives often found themselves sidelined for “promoting dangerous misinformation” or being “anti-science” – even if those people were highly qualified scientists or medical professionals [24, 26, 96, 347].

We argue that, by only considering a single set of scientific narratives, the medical and scientific communities, as well as policymakers, were severely hindered in their ability to critically evaluate the science throughout the pandemic.

We suggest that this narrowing of the range of relevant scientific questions that were tolerated—by (a) the public (b) the media (c) social media platforms (d) the “fact-checking organisations” (e) government officials (f) the pharmaceutical industry and (g) the scientific community itself,—has inadvertently hindered the ability of researchers to engage in open-minded scientific inquiry into these complex scientific problems.

#### Role of the Public

It is known that when people perceive certain behaviours as being associated with the risk of illness or death, their assessment of that behaviour (subconsciously) becomes moralised [348, 349], perhaps even contradicting a sacred value [348, 350, 351]. When we are evaluating an issue from a moral or even sacred perspective, it can be difficult to evaluate it dispassionately, based on purely evidentiary or logical grounds. Moreover, our moral views are often aligned with our political ideology [348, 349], meaning that pre-existing political divisions can aggravate societal divisions on these issues [351, 352].

From the beginning of the pandemic, media coverage, social media and government efforts effectively propagated an escalating sense of fear and panic among many people about the severity and dangers of COVID-19 [161, 344, 353, 354]. Hence, many people found it difficult to listen to different scientific perspectives on COVID-19 policies neutrally and dispassionately. Surveys in mid-2020 found that people with a greater personal fear of contracting COVID-19 were more likely to evaluate COVID-19 policies from a moral or even “sacred values” perspective [351], and that this effect was more pronounced among left-wing participants [351].

Concerns had already been expressed before the pandemic about the tendency for people to self-select into “echo chambers” where people seek information that agrees with their views and avoid dissenting perspectives [355]. Using value-laden terms such as “conspiracy theory” and “the science” to uncritically dismiss or promote particular perspectives seems to have increased this polarisation during the pandemic [356], and appears to have encouraged some people to “scapegoat” others [352]. Meanwhile, those who felt that expressing their opinion might lead to vilification, mocking or harassment, might have increasingly self-censored their views–leading to a “spiral of silence” [357].

#### Role of the Media

Article 19 of the Universal Declaration of Human Rights states that, “Everyone has the right to freedom of opinion and expression; this right includes freedom to hold opinions without interference and to seek, receive and impart information and ideas through any media and regardless of frontiers” [358]. The Global Charter of Ethics for Journalists emphasizes that a journalist’s responsibility to the public in aiding this specific human right “takes precedence over any other responsibility, in particular towards their employers and the public authorities” [359].

On the other hand, in recent years, many in the journalistic profession have revisited these ideals–especially with regard to scientific reporting–because when the public is presented with multiple perspectives they might not reach the same conclusions as the journalists [360, 361]. Hence, many media outlets have begun prioritizing “reliable reporting” (where journalists and editors pre-decide what information the public should be provided with) over more conventional “balanced reporting” (where the journalists try to provide the public with all relevant perspectives) [361]. A consequence of this shift in approaches to scientific reporting by the media is that many editors and journalists have effectively decided to act as gatekeepers of what scientific information to convey to the public. During COVID-19, this was often accompanied with “a tendency to overuse linguistic items implying certainty” in “the science” even as the science in question was repeatedly re-evaluated over time [362].

#### Role of the Social Media Platforms and Internet Search Engines

In recent years, social media platforms and internet search engines have allowed the public the opportunity to research information for themselves and to share with each other information that they think is important. However, this has allowed the public access to a wider range of information than that provided by media outlets, thereby partially undermining the “reliable reporting” project described above. Hence, in recent years, these internet companies have found themselves under increasing pressure to artificially reduce the spread of some information and increase the spread of other information [363, 364].

At the start of COVID-19, platforms such as Facebook and Twitter expressed a willingness to suppress alleged “misinformation” on COVID-19 and promote alleged “accurate information” [28]. However, it is now being increasingly recognised that often the “misinformation” that was being suppressed transpired to be “accurate information” and *vice versa*. In a June 2023 podcast interview, the CEO of Meta (Facebook, Instagram and WhatsApp) admitted that his platforms had been “asked for a bunch of things to be censored that, in retrospect, ended up being more debatable or true” [365]. Decisions over what information to promote versus downrank were often determined by pressure from US government agencies, and often mistakenly involved downranking or censoring genuine scientific opinions [30, 366–368]. At one point, Facebook began penalising users for attempting to share an article published in the British Medical Journal, one of the world’s oldest general medical journals [269].

#### Role of Fact-Checking Organisations

The narratives of popular discourse have increasingly been controlled by fact-checking organizations. These organisations are often partially funded by corporations such as Google and Meta (i.e., Facebook) and purport to identify certain articles as “false,” “misleading” or “missing context”. These fact-checks are then used as justification by social media platforms and media outlets for suppressing the information [269, 369].

These fact-checks can be based on flimsy grounds, misrepresent an article, or even make verifiably false claims, yet the organisations are set up in such a way that successfully appealing the claim is largely impossible [269, 369]. Hence, rather than improving the quality of information available to the public or providing relevant context, fact-checks are effectively no more than a narrative-check, censoring or discrediting scientific opinions that differ from theirs. Not only does this limit the public from being exposed to different scientific opinions, but the reputational damage of sharing information that ends up being “fact-checked” can also lead to self-censorship.

The academic basis for the use of fact-checking for narrative control appears to be based on “inoculation theory” [370, 371]. This is a strategy to minimise the availability of multiple perspectives on certain topics by “pre-bunking” the public with deliberate misrepresentations of compelling arguments into less compelling versions and then countering these strawman versions of the arguments. Analogous to vaccination with a weakened virus, when the public later encounters the original arguments, they will then be “inoculated” into dismissing the arguments without due consideration [370, 371].

#### Role of Government and Scientific Advisory Boards

Ultimately, governments and other policymakers decided national COVID-19 policies, even if they often insisted that they were “following the science” [48, 164–166]. However, there have been conflicting scientific opinions on most aspects of COVID-19 policies, throughout the pandemic. Therefore, they could only have been following *some of* the science at best. Indeed, it seems that governments were often simply following their neighbours [8, 112, 162, 372].

The scientific advisory groups used by governments for deciding COVID-19 policies generally included expertise from only a few relevant aspects of public health and were particularly influenced by modelling groups [48, 70, 165, 166]. Indeed, it has been argued that many of the policies implemented “would be considered unacceptable according to pre-pandemic norms of public health ethics” [373]. Meanwhile, there is concern that government policies were often heavily influenced by scientists and other appointees with potential conflicts of interest [165, 374, 375].

Others have noted that many policies were “authoritarian” and led to “violations of democratic standards” [159, 178], yet did “not correlate with better public health outcomes” [159, 376]. Apparently, many governments used psychological and sociological “nudge” manipulation techniques [377] to promote particular policies and behaviours [69, 220, 354].

Surprisingly, however–despite proclaiming that policy decisions were based on solid and incontrovertible evidence–behind-the-scenes information has revealed that often decisions were made without a concrete rationale [69–72]. The former UK Prime Minister, Rishi Sunak, who was Chancellor of the Exchequer from 2020 to 2022, admitted that UK’s “lockdown” policies were a direct consequence of preliminary model predictions [70]. It was only after the government rejected the modelling-based advice of SAGE, their scientific advisory group, calling for a fourth lockdown in December 2021 that the government realised SAGE’s model predictions were off by a factor of 20. Meanwhile, the chair of SAGE’s modelling committee admitted that they had intentionally excluded the possibility that a new variant might have been less virulent [71].

#### Role of Pharmaceutical Companies

Legg et al. developed a framework for evaluating how and why corporations influence science and the use of science in policy and practice, suggesting that the pharmaceutical industry is potentially using 5 of their identified macro-strategies and 16 of their 19 proposed meso-strategies [378]. However, directly identifying biases specifically caused by the influence of the pharmaceutical industry on the media, social media platforms, government policies and the scientific community is difficult because the industry is so influential that interconnections between all of these different sectors are widespread [231, 379].

Although the direct advertising budget of many pharmaceutical companies on media and social media is only a fraction of their total expenditure budget, this can still represent a large source of advertising revenue for media or social media platforms. For instance, it has been estimated that the pharmaceutical industry spent nearly US$4 billion on TV advertisements in 2021 [380] – potentially biasing the neutrality of those platforms. Recently, it was revealed that Royal Medical Colleges in UK receive large payments from drug and medical devices companies [381]. An analysis of the payments received by French medical doctors from Gilead Science (developers of remdesivir) and their public statements on the potential use of HCQ (a cheap repurposed drug, proposed as an alternative treatment to remdesivir) for COVID-19 treatment during 2020 revealed that those who were most critical of HCQ had received the most funding from Gilead Science [382].

Working relationships between pharmaceutical corporations (including Pfizer, AstraZeneca, Johnson & Johnson, and Moderna), mainstream media (including Thomson Reuters) and tech companies (including Facebook and Google), as outlined by Deruelle, call the impartiality of the social media platforms’ fact-checking initiatives into question [231].

#### Role of the Scientific Community

One of the main mechanisms by which scientists communicate their findings and analysis to other scientists is by publishing their work in peer-reviewed academic journals. However, over the years, the number of published papers has accelerated–especially in subjects where considerable funding is available.

Within just the first year of the pandemic, more than 100,000 COVID-19-related scientific articles were published [383]. Hence, there has clearly been a lot of scientific information, analysis and opinions expressed during the pandemic. This emphasises the erroneous nature of the idea that the science on COVID-19 was ever clear, simple or unambiguous. However, as we will discuss, it appears that many journals ensured research that supported certain scientific narratives was prioritised over research that contradicted those narratives.

Some papers seemed to speed through publication. For example, the paper describing the original RT-PCR test for SARS-CoV-2 was published on 23 January 2020 within less than 48 h of submission [118]. However, other papers have faced extended peer-review processes involving multiple rounds of revisions (often involving resubmitting revised manuscripts to different journals) that have delayed publication for months or even years. As an example the original version of Simandan et al. was first submitted to a journal on 12 December 2020 and had to go through multiple rounds of peer review in multiple journals before being accepted for publication on 11 January 2023 [178]. Given the rapid developments during the pandemic, these delays meant that COVID-19 policy decisions were typically heavily influenced by the papers that were published first. When different perspectives were eventually published months or years later, it was generally too late to make much difference.

For example, both Flaxman et al. and Wood analysed a similar scientific question: How much of an influence did NPIs have on the progression of the first wave of the pandemic (February to May 2020) in Europe? Flaxman et al. (2020) compared the COVID-19 death statistics to a SEIR-modelled counterfactual scenario of what would have occurred in the absence of NPIs and concluded that the NPIs were very effective and that 2.8–3.5 million deaths across 11 countries had been averted [7]. The paper was submitted on 30 March 2020 and accepted for publication in the journal Nature on 22 May 2020. Wood also studied this first wave but found that the wave had already been in decline in the UK before the full implementation of NPIs and in Sweden without NPIs [103]. This study received the first of several rejections in May 2020 and did not appear in print until September 2022.

Given the urgency of developing COVID-19 policies, many researchers took advantage of “pre-print servers” to try and speed up the process of delivering relevant scientific information into the public domain before peer review [383–386]. Some of these preprints influenced COVID-19 policies–especially during the early stages of the pandemic [6, 43, 383, 384]. Some also received media coverage [383, 386]. So, for some researchers, preprints offered a way of conveying their findings to the public and/or policymakers in time for them to be relevant. However, given the large number of COVID-19-related preprints published, unless the researchers had considerable influence on policymakers and/or good access to the media, many of these insights could have been overlooked. Moreover, some researchers have cautioned that many preprint servers have begun using opaque and/or poorly justified reasons for refusing to publish preprints, seemingly based on the conclusions of the manuscripts rather than their scientific merit [385].

An alarming trend we have noticed (and some of us have personally experienced) during the pandemic has been the misuse by some journals of the retraction/withdrawal process. When misapplied, this process can have the effect of both silencing and discrediting researchers for publishing peer-reviewed scientific articles that raise concerns over certain COVID-19 policies. *Until recently*, journal articles were very rarely retracted or withdrawn following publication in a peer-reviewed journal [387, 388]. Moreover, in a 2012 survey of retracted articles, it was found that 67.4% were accused of misconduct (including 43.4% fraud or suspected fraud) and 21.3% due to errors [387]. It was also found that it took an average of 2.5 years from publication until retraction [387].

These historic factors have erroneously convinced most of the scientific community that, if a paper is “retracted” or “withdrawn,” it should be treated as a major warning flag that the researchers involved were either involved in scientific fraud or else made serious scientific errors in their analysis that took several years to identify. However, during COVID-19, several journals began using this previously very rare and cautious tool of retraction/withdrawal as a new form of censorship [24, 388, 389].

Below, we give examples of several high-profile COVID-19 studies that were retracted or withdrawn by a journal without providing an adequate scientific justification or offering the authors a chance to issue a response:

• Two papers by Walach et al. were abruptly retracted with inadequate explanation [390, 391]. One raised concerns about the COVID-19 vaccination programmes, given the low observed efficacy of the COVID-19 vaccines in reducing death or severe illness (absolute risk reduction versus relative risk reduction, see table 2), coupled with the relatively high incidence of severe adverse events (as of June 2021). The other raised concerns over COVID-19 policies promoting the prolonged use of face masks by children in schools. Revised versions of both articles were subsequently published in different journals [389, 392, 393], and other researchers have reached similar findings [342, 394]. None of the journals gave sufficiently robust reasons why the studies should be retracted, and they refused to publish the authors’ responses.
• Kostoff et al. (2022) asked whether COVID-19 vaccination was suitable for children, given the very low incidence of severe COVID-19 among children and the potential risks of adverse reactions [395]. This paper was abruptly retracted because one of the co-authors of Kostoff et al. (2022) was an editor of the journal at the time and another editor had handled the manuscript. This meant that at least two of the journal’s editors believed that the paper was publishable. Yet, a third editor disagreed and retracted the manuscript, offering only a vague and debateable rationale for doing so. Multiple independent studies have raised concerns similar to those raised in the now retracted paper [301, 332–334, 342].
• Savaris et al. (2021) statistically evaluated the effectiveness of “stay-at-home” measures, based on publicly available datasets, and found that these policies had no effect on COVID-19 mortality in more than 98% of the regions studied. Two comments were published on this article that disputed the suitability of the statistical approach used [396, 397]. Tellingly, neither comment carried out a counter-analysis of the dataset or provided an alternative statistical approach that they believed would be suitable. Despite the fact that the existence of these comments was obvious on the journal page, the journal retracted the manuscript [398]. As discussed earlier, multiple studies have since confirmed Savaris et al.’s key findings [64, 81, 96, 103, 105, 137–139, 143].
• Rose and McCullough (2021) presented an analysis of the US VAERS data that highlighted a concerning safety signal for the COVID-19 vaccines, showing a substantial increase in myocarditis rates among young adults, but the article was abruptly withdrawn after publication [399]. The withdrawal note simply states, “This article has been withdrawn at the request of the author(s) and/or editor.” Neither author was provided with a scientific justification for this decision and, as discussed earlier, the increased incidence of myocarditis among young adults following COVID-19 vaccination has since been confirmed [296, 298, 299, 301–303].

## Conclusion and Recommendations

### On the Use of Mathematical and Computer Models for Policy Advice

Conclusion 1: Mathematical and computer models can be very powerful tools for evaluating the implications of our current scientific understanding of policy-relevant issues, including epidemic modelling. However, the model output is a consequence of the input assumptions, approximations and data. In the case of COVID-19, it is now apparent that the COVID-19 models that were highly influential on public-health policies (especially in the beginning of the pandemic) were not accurately modelling the real-world progression of the COVID-19 pandemic.

Recommendation 1: Models should be used as a tool to supplement, not replace empirical analysis. While model projections can potentially offer some speculative scenarios for consideration by policymakers–especially in the early months of a pandemic–they should be treated with considerable scepticism. Their relevance and suitability should be continually revisited and, crucially, empirically reassessed as time elapses.

### On the Use of Non-Pharmaceutical Interventions (NPIs) During Pandemics

Conclusion 2: While many of the NPIs implemented during the pandemic had some theoretical justification on mechanistic grounds and support from model-based assessments, many other studies have shown empirically that the NPIs were much less effective than thought, or not effective at all. Meanwhile, the NPIs have also had many unintended adverse public health impacts.

Recommendation 2: If NPIs are ever to be considered again, holistic health impact assessments are essential. Also, methods for objectively assessing their effectiveness should be continuously, empirically scrutinised.

### On the Use of Pharmaceutical Interventions (PIs) During Pandemics

The authors of this essay have different views on the relative safety and effectiveness of both classes of PIs. Nonetheless, by now the following is clear to us:

Conclusion 3: The discouragement of research into the identification of potential treatments using cheap repurposed drugs is disquieting. In particular, two of the candidates (HCQ and ivermectin) had both been widely used for decades before the pandemic and had well-established safety profiles. Therefore, even if neither candidate had any effectiveness, we find the brisk dismissal of research into their potential use disturbing. Conversely, if they were even partially effective at reducing the severity of COVID-19 infections, then the blanket suppression of their use seems even more concerning.

Recommendation 3: Research into the development of potential treatments using generic repurposed drugs with well-established safety profiles should have been encouraged rather than discouraged. We should ensure that responses to future pandemics will welcome rather than oppose such research.

Conclusion 4: It is now apparent that the confident claims of both safety and efficacy/effectiveness made about the COVID-19 vaccines at the start of the vaccination programmes were overly optimistic. These vaccines do not completely preclude infection, or transmission. Some analyses still suggest that the vaccines might reduce the severity of infection, but the evidence for this remains contentious. Meanwhile, it is now clear that the incidence of serious adverse reactions is greater than initially acknowledged. Having reviewed the literature, in hindsight, many researchers had actually been warning of each of the above points–yet their cautions were criticised, penalized, or ignored rather than taken on board.

Recommendation 4: Researchers should be encouraged to critically evaluate claims that a particular vaccine is safe and effective without the fear of potentially being labelled as anti-vax or anti-science, if their research findings reveal any negative results.

Conclusion 5: The original justification for carrying out *population-wide* vaccination programmes was based on the explicit assumption that vaccinating the population past the theoretical “herd immunity threshold” would substantially reduce viral transmission [16]. Based on this, many governments introduced vaccine mandates or other coercive measures to maximise the vaccine uptake rates [16, 342]. However, even though that justification was invalidated early in the vaccination programmes, the programmes (and mandates) continued for many countries until late 2022/early 2023. Given the fact that the characteristics of persons at risk of severe COVID-19 were well-defined, a population-wide vaccination programme was unnecessary. The realisation that there are non-trivial risks of serious adverse reactions associated with many of the vaccines, especially the mRNA and DNA vaccines, turned the vaccination of people at low risk of severe COVID-19 into an unnecessary public-health risk. The use of mandates and other measures to nudge people into being vaccinated also raises considerable ethical and moral problems [16, 284, 342, 400, 401].

Recommendation 5: Future vaccination programmes should involve a more thorough evaluation of the safety and efficacy/effectiveness of the vaccines in relevant subgroups; if still deemed necessary, programmes should be based on genuine voluntary consent.

Conclusion 6: The autonomy of both patients and their doctors in deciding the most suitable healthcare pathways for each individual was heavily undermined by nationwide health policies that seem to have been heavily influenced by the pharmaceutical industry. Patients or doctors who expressed an interest in the use of repurposed drugs were often refused the opportunity. In contrast, patients or doctors who expressed any concern about the suitability of the available COVID-19 vaccines often faced considerable adversity or hostility. Meanwhile, patients who reported adverse reactions following COVID-19 vaccination experienced “medical gaslighting” and often seemed to encounter a blind spot from authorities when it came to considering the possibility it might have been associated with the vaccines [215, 218].

Recommendation 6: Hippocrates proposed that the combatting of disease should involve an individual collaboration between physician and patient. While we appreciate that pharmaceutical companies produce products for mass usage and that national health services often design policies at a national level, it is of paramount importance that we ensure that patients and physicians are allowed to work together to develop personal healthcare pathways individually designed for the circumstances of each patient.

### The Inadvertent Suppression of Valid Scientific Perspectives as a Side Effect of Efforts to Reduce the Spread of Misinformation

Conclusion 7: In a misguided attempt to reduce “the spread of misinformation”, media outlets, social media platforms, government agencies and scientific journals have severely restricted access to valuable scientific information and severely hampered the ability of everyone to have informed discussions of complex, multifaceted problems associated with COVID-19.

Recommendation 7: In our opinion, the best antidote to bad ideas is to counter them with better ideas. Censorship of different scientific opinions does not lead to better scientific opinions–it leads to weaker scientific conclusions. Scientific freedom should be cherished. If not, the costs to humanity may be very high.
